# Effects of Pretreatment on the Isotopic Composition of Structural Carbonate in Ray‐Finned Fish Teeth

**DOI:** 10.1002/rcm.70200

**Published:** 2026-10-22

**Authors:** Toshikazu Furuya, Kazuki Yokouchi, Taiga Okumura, Kotaro Shirai

**Affiliations:** ^1^ Atmosphere and Ocean Research Institute The University of Tokyo Kashiwa Chiba Japan; ^2^ Fisheries Resources Institute Japan Fisheries Research and Education Agency Yokohama Kanagawa Japan; ^3^ Faculty of Education and Integrated Arts and Sciences Waseda University Tokyo Tokyo Japan

**Keywords:** dentin, fish tooth, isotope, pretreatment, structural carbonate

## Abstract

**Rationale:**

The carbon and oxygen stable isotope ratios (δ^13^C and δ^18^O) of carbonate contained in fish tooth dentin have the potential to offer insights into environmental and ecological conditions, making them promising targets for analysis in fossil specimens. However, the tooth dentin of extant specimens contains significant amounts of organic matter that can decompose or produce gases that interfere with isotope measurements. Therefore, a well‐developed pretreatment protocol for removing organic components prior to isotopic analysis is essential.

**Methods:**

In this study, we developed pretreatment methods for removing organic matter to enable the isotopic analysis of structural carbonates in tooth dentin. Using teeth of the half‐smooth golden pufferfish ( *Lagocephalus spadiceus* ) as specimens, we applied a total of eight physical and chemical pretreatment protocols and evaluated their effects on carbonate isotopic composition.

**Results:**

Treatments with neutral or alkaline pancreatin solutions effectively removed organic matter without altering the δ^13^C values of the carbonate. In contrast, no pretreatment method was identified that kept δ^18^O values entirely unaffected. However, treatment with the neutral pancreatin solution resulted in sufficient removal of organic matter and only minimal, consistent changes in δ^18^O.

**Conclusions:**

Treatments with neutral or alkaline pancreatin solutions are recommended for the analysis of δ^13^C in carbonate within dentin. Although no method for ideal organic matter removal without changing δ^18^O values was identified, treatment with neutral pancreatin solution is recommended as the second‐best option. Because of the small and consistent change in δ^18^O, it is possible to correct for δ^18^O alterations induced by pretreatment.

## 1 Introduction

Ray‐finned fish comprise half the species of vertebrates [1], and their habitats are highly diverse. Although ray‐finned fish (*Actinopterygii*) are one of the most prevalent taxa in aquatic ecosystems, their evolution remains largely unknown [2]. Their nuclear DNA, mitochondrial DNA, and fossil abundance in sediments revealed that they have increased species and populations rapidly since the Cenozoic era [3, 4]. Some researchers have assumed that migration ecology plays an important role in the evolution [5, 6]. Despite the rapid radiation of ray‐finned fishes during the early Cenozoic, the fossil record preserving direct evidence of the evolutionary histories of each clade remains sparse [3], limiting the reconstruction of their evolutionary history. In addition, divergence time estimates derived from molecular data exhibit uncertainties that exceed the temporal scale at which speciation is believed to have occurred, thereby compromising the accuracy of the phylogenetic inferences. Consequently, the evolutionary history and diversification of ray‐finned fishes remain largely unknown [2].

Fish fossils provide powerful clues for understanding their evolution. Fossils are direct evidence of existence and can be used to reconstruct the mysterious lineage of fish [1, 3]. Fish debris, particularly small fish fossils, such as fish teeth and scales, is relatively common in pelagic clays sedimented after the Cenozoic era [4]. At the foot of seamounts or ocean plateaus, they sometimes contain up to 30 vol% of fish debris in all sedimentary particles [7]. The number of ray‐finned fish teeth contained in fish debris is large, 5–100 times more than the scales of elasmobranchs [4]. Because of their relative abundance, fossil teeth are important for understanding the ecology of past ray‐finned fish.

The isotope values of structural carbonates in fossil teeth provide environmental, ecological, and physiological information on vertebrates, including fish [8, 9, 10]. In the crystal structure of hydroxyapatite, carbonate ions are substituted for hydroxyl (OH^−^) and phosphate (PO_4_^3−^) ions [11, 12]. The stable carbon isotope ratio (δ^13^C) of structural carbonate records metabolism, trophic level, and δ^13^C of dissolved inorganic carbon in ambient water [8, 13]. In addition, the stable oxygen isotope ratio (δ^18^O) of structural carbonate records the temperature and salinity of ambient water [9, 10, 14]. These hypotheses are supported by isotopic analysis of structural carbonate in bone [15, 16] and synthetic apatite [17]. In particular, the mechanisms of isotope fractionation and incorporation in terms of taxa, ecology, and physiology have been relatively well elucidated in modern teeth of terrestrial mammals [8, 18]. However, these mechanisms have not been elucidated for fish teeth, despite diverse taxa, habitats, and ecology of fish.

Although modern samples are key to understanding the mechanism of isotope fractionation and incorporation, modern tooth samples contain a relatively large amount of organic matter, in particular in dentin, which challenges isotope analysis of these modern samples in several aspects. First, organic matter in wet samples facilitates bacterial flourishing, producing organic acids [19] that dissolve and deteriorate tooth samples [20]. Organic matter may furthermore prevent samples from reacting with phosphoric acid, which is needed to obtain sample CO_2_ gas for isotope analysis [21]. In addition, volatile organic matter from soft tissue harms the gas chromatographic column, or high‐vacuum and high‐voltage parts in the isotope ratio mass spectrometer [22]. In addition, volatile organic matter and CO_2_ gas generated by the decomposition of organic matter may contaminate the CO_2_ gas derived from structural carbonates and change the isotope ratios obtained from the analysis [23]. Therefore, organic matter should be removed beforehand by pretreatment for sample preservation and isotopic analysis.

When removing organic matter from teeth, the carbonate isotopic composition can change with secondary carbonate formation depending on the method [24], and the degree of change may vary depending on the microstructural elements of the tooth [25, 26]. Fish teeth are often composed of enameloid caps and dentin elsewhere [27, 28]. As the entire tooth size is often smaller than 1 mm [7, 29], dentin can be used as a target for isotopic analysis to ensure a sufficient amount of sample. Although enameloid contains less organic matter and water (approximately 4 wt.%) dentin contains about 30 wt.% organic matter and water [30]. A method for removing organic matter from enameloid without altering the isotopic composition of structural carbonate has been proposed [9, 13], but no such method has been suggested for dentin [25, 26], despite dentin comprising the main proportion of teeth and being particularly important for modern applications.

This study aims to establish an organic matter removal method for isotopic analysis of structural carbonates in dentin. To evaluate the effects of organic matter removal on the isotopic composition of structural carbonate, we determined the secondary formation of carbonate and the changes in isotopic composition induced by pretreatment.

## 2 Materials and Methods

### 2.1 Materials

The teeth of half‐smooth golden pufferfish ( *Lagocephalus spadiceus* (Richardson, 1845)) were analyzed. The teeth were large (more than 1 cm in size), providing a sufficient sample for analysis. The 39 specimens were caught in Ise Bay, near Aichi Prefecture, and immediately frozen at −25°C. Information on these specimens is provided in Table S1, Figure S1, and Figure S3 [31, 32]. Thin layers of enameloid overlap in the teeth of the specimens were embedded in the dentin [33, 34]. The enameloid layers in the teeth of the specimens were thin (< 1 μm) and few (approximately 4 in number) (Figure S2), so the volume of enameloid can be ignored in terms of the effect of the dental microstructure. Four teeth were located on the left and right sides of both the upper and lower jaws in the specimens, and all the teeth in each specimen were extracted as samples (*n* = 152). In addition, the vertebrae, which differ in microstructure from dentin, were analyzed to test whether the effect of chemical treatment on the isotopic composition of structural carbonate is different from that of dentin. For each pretreatment, three vertebrae samples were randomly extracted from each specimen. Information on vertebrate samples (*n* = 123) is provided in Table S3.

### 2.2 Pretreatments

Each whole tooth (0.22–0.27 g by dry weight) and whole vertebra (0.05–0.06 g by dry weight) was subjected to the following pretreatments.

#### 2.2.1 Physical Cleaning

Soft tissues, such as muscles attached to the teeth, were removed as much as possible using tweezers.

#### 2.2.2 Soft Tissue Fixation

Organic solvents such as ethanol and acetone fix soft tissues and prevent sample decay [35, 36]. Samples were soaked in 10 mL of 99.5% ethanol or 99% acetone (Fujifilm Wako Pure Chemical Co., Osaka, Japan) for 1 day.

#### 2.2.3 Oxidation of Organic Matter

H_2_O_2_ aq. and NaClO aq. oxidize and remove adherent organic tissue, which is commonly used for the removal of organic matter for elemental and isotopic analysis of biogenic carbonates and phosphates [26, 37]. The samples were soaked in 10 mL of a 30% H_2_O_2_ aq. (Fujifilm Wako Pure Chemical Co., Osaka, Japan) for 1 day, or 10 mL of 2% NaClO aq. (prepared from 5% NaClO aq., Hayashi Pure Chemical Ind., Osaka, Japan) for 3 days. Reagent concentrations and reaction times were set as described by Koch et al. [26].

#### 2.2.4 Hydrolysis of Organic Matter

Alkaline solutions, such as NaOH aq., hydrolyze ester, and peptide bonds that constitute chitin, proteins, and lipids [38, 39]. Samples were soaked in 10 mL of 0.1 mol/L NaOH aq. (Merck KGaA, Darmstadt, Germany) for 1 day. Reagent concentration was set as described by Chung et al. [16]. Digestive enzymes also hydrolyze proteins, lipids, and carbohydrates [40, 41]. Thus, adherent tissues in each sample were hydrolyzed with 10 mL of 1% pancreatin solution (prepared using pancreatin powder from porcine pancreas, Tokyo Chemical Industry Co., Tokyo, Japan) at 40°C for 1 day. In addition, an alkaline pancreatin solution was prepared by dissolving borax (Fujifilm Wako Pure Chemicals Co., Osaka, Japan), and the pH was maintained at 9 to enhance the pancreatin activity. Some samples were soaked in the alkaline pancreatin solution at 40°C for 1 day.

One of the two upper and lower teeth of each individual was subjected to physical cleaning and the other was subjected to one of the chemical treatments described above as a pair (Table S1). Of the vertebral samples derived from the same individual, two or three were subjected to physical cleaning, and another two or three were subjected to one of the chemical treatments described above (Table S3). After pretreatment, the samples were rinsed six times with Milli‐Q water, and the pH of the washing solution was confirmed to be between 6 and 7. The rinsed samples were dried for 1 day in a freeze dryer, FDU‐1200 (Tokyo Rikakikai Co., Tokyo, Japan). The dried samples were crushed using a freeze‐crush device, Auto‐mill TK‐AM7 (Tokken Inc., Chiba, Japan) (1800 rpm, 60 s), and approximately 2.2 mg of the sample powder was obtained for isotope analysis.

### 2.3 Isotope Analysis

Phosphoric acid was added to the tooth sample powders, and the tooth sample powders were reacted completely at 72°C. The CO_2_ produced was analyzed for isotopic composition and abundance of structural carbonate using a Delta V Plus equipped with a Finnigan GasBench II (Thermo Fisher Scientific, Bremen, Germany) at the Atmosphere and Ocean Research Institute, the University of Tokyo. The measurements were performed relative to the composition of a CO_2_ reference gas. The isotope values were calibrated against NBS 19 (National Bureau of Standards, USA), and JCp‐1 and JCt‐1 (National Institute of Advanced Industrial Science and Technology, Japan) that were analyzed together with the samples as reference materials. The long‐term reproducibility (1 SD) was 0.05‰–0.11‰ for δ^13^C, and 0.06‰–0.44‰ for δ^18^O (Table S4, *n* = 177). Isotope values were calculated based on delta notation and compared with the standard Vienna Pee Dee Belemnite values:

$$\delta^{13C}\;\text{or}\;\delta^{18O}=\left(\frac{R_{\text{sample}}}{R_{\text{standard}}}-1\right)\times 1000‰$$

where *R* is the ^18^O/^16^O or ^13^C/^12^C ratio of the sample or standard. Details of the analytical settings were reported by Shirai et al. [42] and Chung et al. [16].

In order to determine the abundance of structural carbonate in our samples, we plotted a calibration curve of the amplitude of the sample gas at mass 46 (^12^C^16^O^18^O isotopologue) against the weight of the reference material NBS 19 (*n* = 33). We then converted the signal amplitude of the sample to the weight of the structural carbonates assuming a 100% yield for the carbonate conversion to CO_2_. The estimated weights of the structural carbonates were divided by the original weight of the powder to determine the percentage abundance of structural carbonates in teeth or vertebrates.

To evaluate the potential influence of residual organic matter, dry muscle sample powders were also analyzed. First, muscle tissues from several individuals (see Table S2) were freeze‐dried under vacuum, powdered, and analyzed using the same mass spectrometer and analytical procedure as the tooth samples. Next, muscle sample powders from given individuals were mixed with the corresponding tooth sample powders from these individuals, which had been subjected to each of the chemical treatments (see Table S2).

### 2.4 Statistical Analysis

Statistical analyses were conducted to evaluate the changes in carbonate content (ΔCO_3Tooth_ for tooth samples and ΔCO_3Vert_ for vertebrae samples) and isotope composition (Δ^13^C_Tooth_ and Δ^18^O_Tooth_ for tooth samples and Δ^13^C_Vert_ and Δ^18^O_Vert_ for vertebrae samples) caused by each chemical treatment. The carbonate content and isotopic composition of the physically cleaned samples were assumed to represent the primary values and were used as references for the evaluation. To evaluate the influence of adhesive organic material on the analytical results of samples subjected to physical cleaning, the influence of potential contaminant gases on isotopic analysis was monitored. In addition, the carbonate content (wt.%) was checked to evaluate the CO_2_ gas released from other contaminants. In addition, the isotopic composition of the tooth samples and the corresponding tooth samples mixed with the muscle powder were compared using a Wilcoxon matched‐pairs signed‐rank test (*n* = 42, See Table S2 and Section 4). Next, for the upper and lower teeth, the values from physically cleaned and chemically treated samples from the same individual were compared. In enamel, no isotopic differences are observed between the left and right teeth [43], and it is therefore likely that dentin also shows consistent isotopic compositions and carbonate weight percentages between the sides. In this study, one tooth from either the upper or lower jaw of the same individual was subjected to physical cleaning, whereas the other was subjected to chemical treatment. Thus, the original carbonate content and isotopic composition of the physically cleaned and chemically treated teeth, both derived from the same individual, were expected to match. Because of this match and the small sample size of each treatment, the changes in carbonate content (wt.%) and isotopic composition were assessed using a Wilcoxon Matched‐Pairs Signed‐Rank Test [44] (*n* = 152). In contrast, vertebral samples may exhibit intraindividual variation in primary isotopic composition [16], and the number of chemically treated samples was also limited. Therefore, changes in the carbonate content and isotopic composition of vertebral samples were evaluated using analysis of variance (ANOVA) with aligned rank transform (ART) [45] (*n* = 123). All analyses were performed using R software (R version 4.4.1) [46], and the ARTool package was used for ART [47]. The *wilcox.test* function was used for the Wilcoxon matched‐pairs signed‐rank test, and the *anova* function was used for the ANOVA with ART.

### 2.5 X‐Ray Diffraction (XRD) Analysis

During diagenesis in nature, hydroxyapatite, the principal mineral phase of teeth, can be transformed into fluorapatite or acquire secondary mineral phases such as carbonate apatite through elemental substitution and other diagenetic processes [48]. In this study, we obtained the XRD patterns of the powdered samples (*n* = 15) to evaluate the change in the mineral phase due to each chemical treatment. For the analysis, tooth powders subjected to each pretreatment were randomly selected (one or a few subsamples per pretreatment), and their mineral phases were examined by XRD. XRD measurements were conducted using a RINT‐Ultima^+^ diffractometer equipped with a D/teX Ultra silicon strip detector (Rigaku, Tokyo, Japan) and Cu K*α* radiation emitted at 40 kV and 30 mA. The samples were ground into powder in an agate mortar and were loaded on a glass sample holder. Cu K*β* was eliminated by a Ni thin foil. A 1° divergence slit, a 10 mm mask to confine the beam width, and a 10‐mm antiscatter slit were applied. The data were collected at 0.02° (2*θ*) intervals with a scan rate of 1°/min.

## 3 Results

A comparison of the appearance, amount (wt.%), and isotopic composition of structural carbonate, and XRD patterns of the samples that experienced physical cleaning and those that experienced chemical treatments is presented below.

### 3.1 Appearance of Fish Teeth After Treatments

Much organic matter, such as adhesive muscles, remained after treatment with physical cleaning, ethanol, acetone, and NaOH aq. We will discuss later the influence of organic matter on the analytical results, especially the samples subjected to physical cleaning, based on the effects of potential contaminant gases on isotope analysis and measured carbonate content (See Section 4). In contrast, all adhesive muscles were removed from the tooth and vertebrae samples after treatments with H_2_O_2_ aq., NaClO aq., neutral, and alkaline pancreatin solutions (Figure 1, Figure S4, and Figure S5).

**FIGURE 1:**
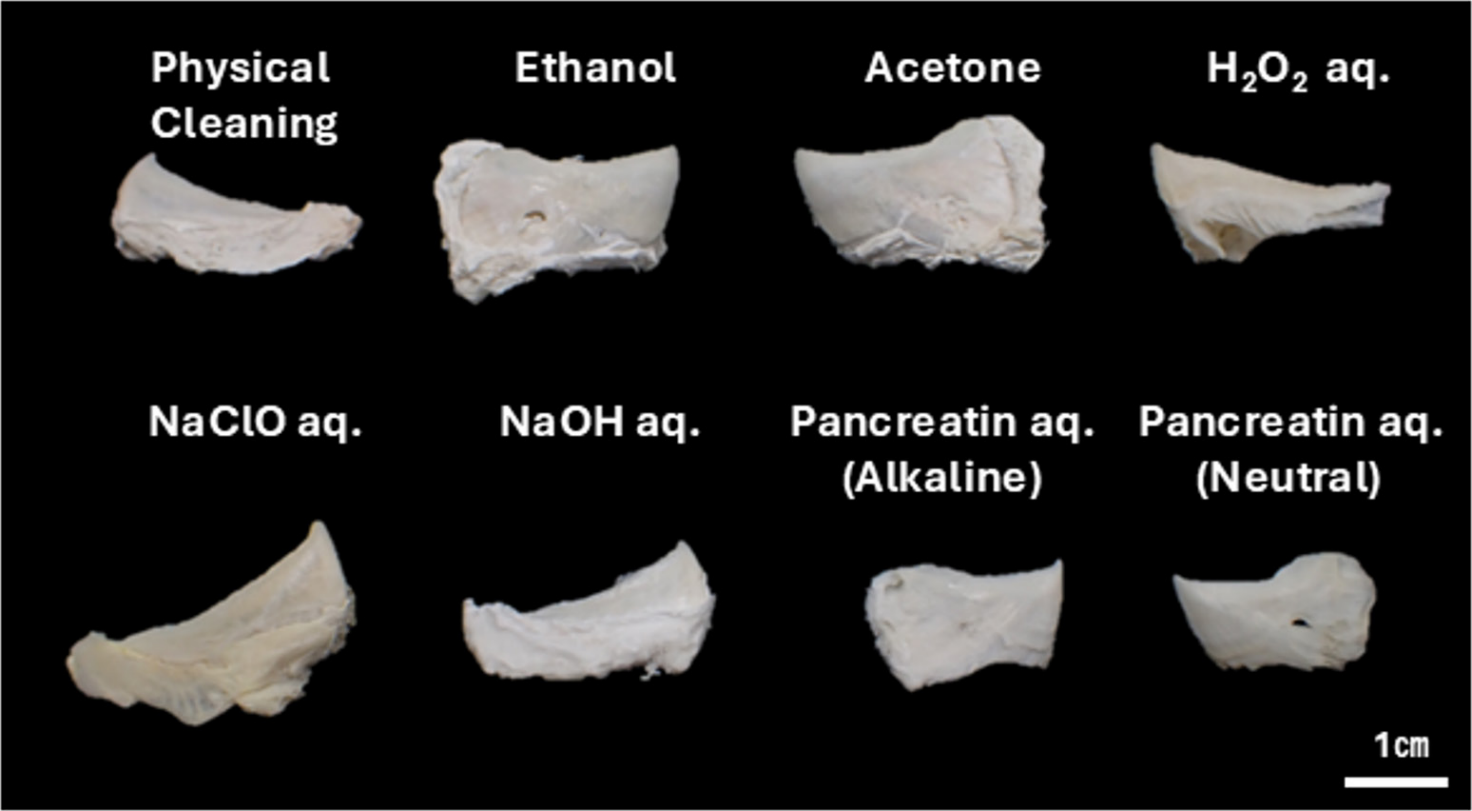
The appearance of tooth samples after pretreatment. The teeth shown here have undergone each pretreatment. All samples were photographed from the labial side. Both left and right, upper and lower jaw teeth are included.

### 3.2 Carbonate Percentage

The carbonate content (wt.%) was not affected by treatments with organic solvents or acidic and neutral solutions but increased significantly by treatments with strong alkaline solutions. Also, no CO_2_ peak was detected from the muscle sample powders in the isotope analysis. The carbonate content of samples treated with ethanol, acetone, H_2_O_2_ aq., or neutral pancreatin solution did not differ significantly or showed small changes from that of samples subjected to physical cleaning, for both teeth and vertebrae (See Figure 2, Figure S6, and Table 1). In contrast, samples treated with NaClO aq. showed a significantly higher carbonate content compared to physically cleaned samples (ΔCO_3Tooth_ = 2.97 ± 1.30 wt.%, *p* < 0.01, *n* = 16; ΔCO_3Vert_ = 8.97 ± 0.72 wt.%, *p* < 0.001, *n* = 25) (Figure 2). Similarly, samples treated with NaOH aq. also exhibited a significant increase in carbonate content relative to physically cleaned samples (ΔCO_3Tooth_ = 1.15 ± 0.37 wt.%, *p* < 0.01, *n* = 20; ΔCO_3Vert_ = 1.68 ± 0.35 wt.%, *p* < 0.001, *n* = 28) (Figure 2). No significant change in carbonate content was observed in tooth samples treated with an alkaline pancreatin solution (ΔCO_3Tooth_ = 0.09 ± 0.33 wt.%, *p* = 0.56, *n* = 20), whereas a significant increase was found in vertebra (ΔCO_3Vert_ = 1.01 ± 0.10 wt.%, *p* < 0.001, *n* = 29) (Figure 2).

**FIGURE 2:**
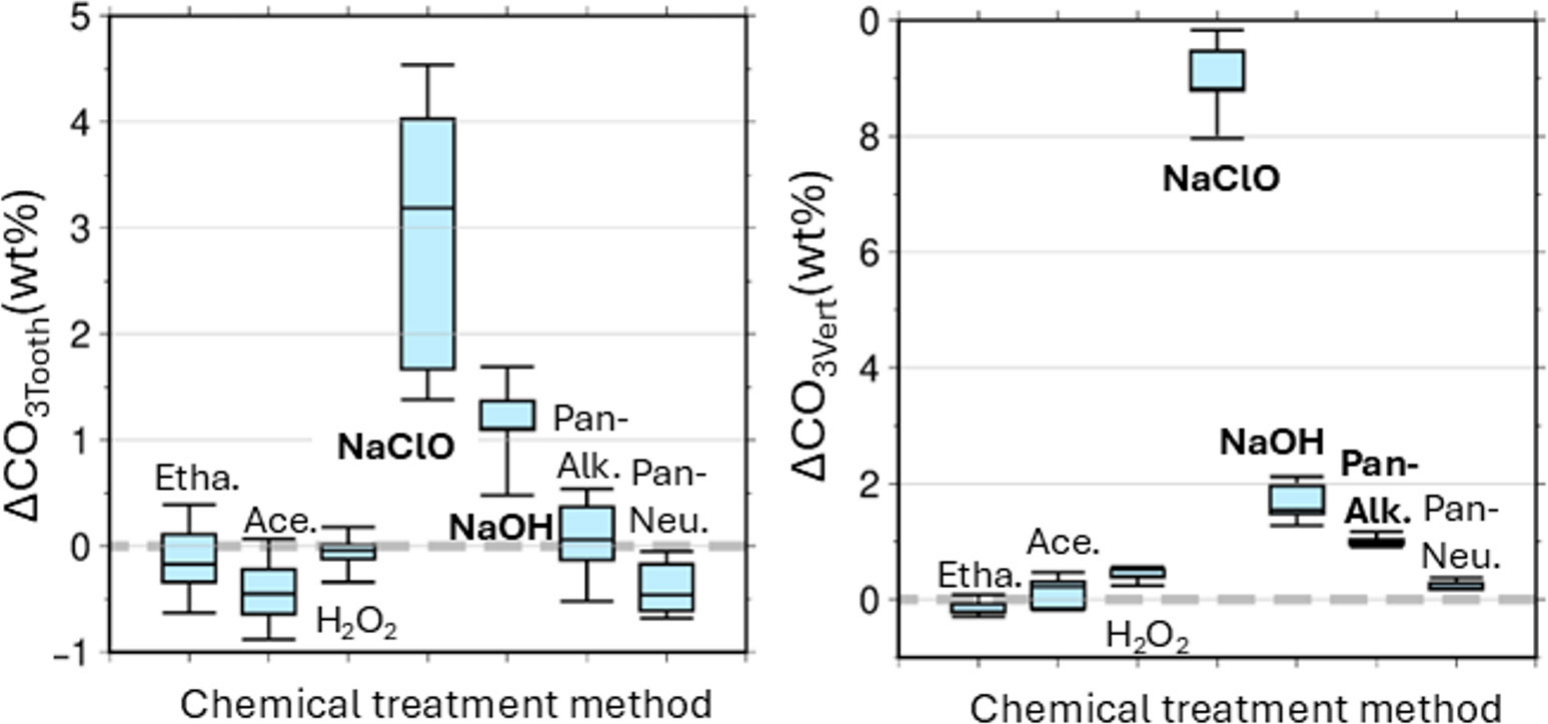
Changes in carbonate content caused by chemical treatment. Changes in the carbonate content of tooth (left graph) and vertebrae (right graph) samples after chemical treatment with each reagent are shown as box plots. The reagent used for each treatment is indicated near the corresponding plot. (Etha.: Ethanol, Ace: Acetone, H_2_O_2_: H_2_O_2_ aq., NaClO: NaClO aq., NaOH: NaOH aq., Pan‐Alk.: Alkaline Pancreatin aq., Pan‐Neu.: Neutral Pancreatin aq.) A value of 0 on the vertical axis indicates no change in carbonate content. Treatments that caused statistically significant increases exceeding 1% are shown in bold.

**TABLE 1:** Changes in carbonate content (wt.%) and isotopic composition.

	Method 1: ethanol	Method 2: acetone	Method 3:H_2_O_2_ aq.	Method 4:NaClO aq.	Method 5:NaOH aq.	Method 6: alkaline pancreatin aq.	Method 7: neutral pancreatin aq.
ΔCO_3Tooth_(wt%)	−0.14 ± 0.32 (*p* = 0.24) (*n* = 10)	−0.43 ± 0.30 (*p* = 0.0039) (*n* = 10)	−0.07 ± 0.15 (*p* = 0.10) (*n* = 14)	+2.97 ± 1.30 (*p* = 0.0078) (*n* = 8)	+1.15 ± 0.37 (*p* = 0.0020) (*n* = 10)	+0.09 ± 0.33 (*p* = 0.56) (*n* = 10)	−0.40 ± 0.24 (*p* = 0.0020) (*n* = 10)
ΔCO_3Vert_(wt%)	−0.15 ± 0.15 (*p* = 0.0026) (*n* = 14)	+0.13 ± 0.29 (*p* = 0.068) (*n* = 11)	+0.42 ± 0.13 (*p* = 0.000011) (*n* = 12)	+8.97 ± 0.72 (*p* = 0.000014) (*n* = 11)	+1.68 ± 0.35 (*p* = 7.3E‐07) (*n* = 14)	+1.01 ± 0.10 (*p* = 3.8E‐07) (*n* = 14)	+0.24 ± 0.09 (*p* = 0.00032) (*n* = 15)
Δ^13^C_Tooth_(‰VPDB)	−0.39 ± 0.49 (*p* = 0.027) (*n* = 10)	+0.09 ± 0.28 (*p* = 0.19) (*n* = 10)	−1.15 ± 0.91 (*p* = 0.0021) (*n* = 14)	−0.53 ± 0.75 (*p* = 0.034) (*n* = 12)	−3.22 ± 0.32 (*p* = 0.0020) (*n* = 10)	−0.26 ± 0.19 (*p* = 0.0020) (*n* = 10)	−0.10 ± 0.27 (*p* = 0.23) (*n* = 10)
Δ^13^C_Vert_(‰VPDB)	−0.05 ± 0.15 (*p* = 0.53) (*n* = 14)	+0.24 ± 0.62 (*p* = 0.0032) (*n* = 11)	−1.28 ± 0.18 (*p* = 7.8E06) (*n* = 12)	−0.16 ± 0.41 (*p* = 0.16) (*n* = 14)	−2.06 ± 0.51 (*p* = 6.9E‐07) (*n* = 14)	−1.26 ± 0.23 (*p* = 3.9E‐07) (*n* = 14)	−0.11 ± 0.47 (*p* = 0.24) (*n* = 15)
Δ^18^O_Tooth_(‰VPDB)	−0.27 ± 0.68 (*p* = 0.43) (*n* = 10)	+0.08 ± 0.39 (*p* = 0.32) (*n* = 10)	−0.35 ± 0.47 (*p* = 0.013) (*n* = 14)	−3.56 ± 1.28 (*p* = 0.00049) (*n* = 12)	−2.32 ± 0.42 (*p* = 0.0020) (*n* = 10)	−1.11 ± 0.49 (*p* = 0.0020) (*n* = 10)	−0.95 ± 0.29 (*p* = 0.0020) (*n* = 10)
Δ^18^O_Vert_(‰VPDB)	−0.10 ± 0.53 (*p* = 0.81) (*n* = 14)	+0.87 ± 0.83 (*p* = 0.00067) (*n* = 11)	−0.87 ± 0.26 (*p* = 3.1E‐05) (*n* = 12)	−0.26 ± 1.20 (*p* = 0.34) (*n* = 14)	−4.25 ± 1.32 (*p* = 6.4E‐07) (*n* = 14)	−1.92 ± 0.36 (*p* = 3.7E‐07) (*n* = 14)	−0.73 ± 0.41 (*p* = 1.5E‐07) (*n* = 15)

*Note:* Changes in the carbonate content, δ^13^C values, δ^18^O values of tooth and vertebrae samples after chemical treatment with each reagent are shown in tables. The values are presented as mean ± 1 SD.

### 3.3 δ^13^C Values

δ^13^C values (‰VPDB) of the tooth samples were not affected by the treatments with organic solvents or neutral solutions, but decreased substantially by treatments with strong acidic and alkaline solutions. The addition of the muscle samples also did not change δ^13^C values (the difference is −0.04‰, *p* > 0.05, *n* = 42, Table S2). The δ^13^C of carbonate in the samples treated with ethanol, acetone, NaClO aq., or neutral pancreatin solution did not differ significantly from that in samples subjected to physical cleaning, of both teeth and vertebrae (See Figure 3, Figure S7, and Table 1). In contrast, samples treated with H_2_O_2_ aq. showed a significantly lower δ^13^C compared to physically cleaned samples (Δ^13^C_Tooth_ = −1.15‰ ± 0.91‰, *p* < 0.01, *n* = 28; Δ^13^C_Vert_ = −1.28‰ ± 0.18‰, *p* < 0.001, *n* = 26) (Figure 3). Similarly, the samples treated with NaOH aq. also exhibited a significant decrease in δ^13^C relative to physically cleaned samples (Δ^13^C_Tooth_ = −3.22‰ ± 0.32‰, *p* < 0.01, *n* = 20; Δ^13^C_Vert_ = −2.06‰ ± 0.51‰, *p* < 0.001, *n* = 28) (Figure 3). In samples treated with an alkaline pancreatin solution, small change in δ^13^C was observed in tooth (Δ^13^C_Tooth_ = −0.26‰ ± 0.19‰, *p* < 0.01, *n* = 20), whereas a significant decrease was found in vertebra (Δ^13^C_Vert_ = −1.26‰ ± 0.23‰, *p* < 0.001, *n* = 29) (Figure 3).

**FIGURE 3:**
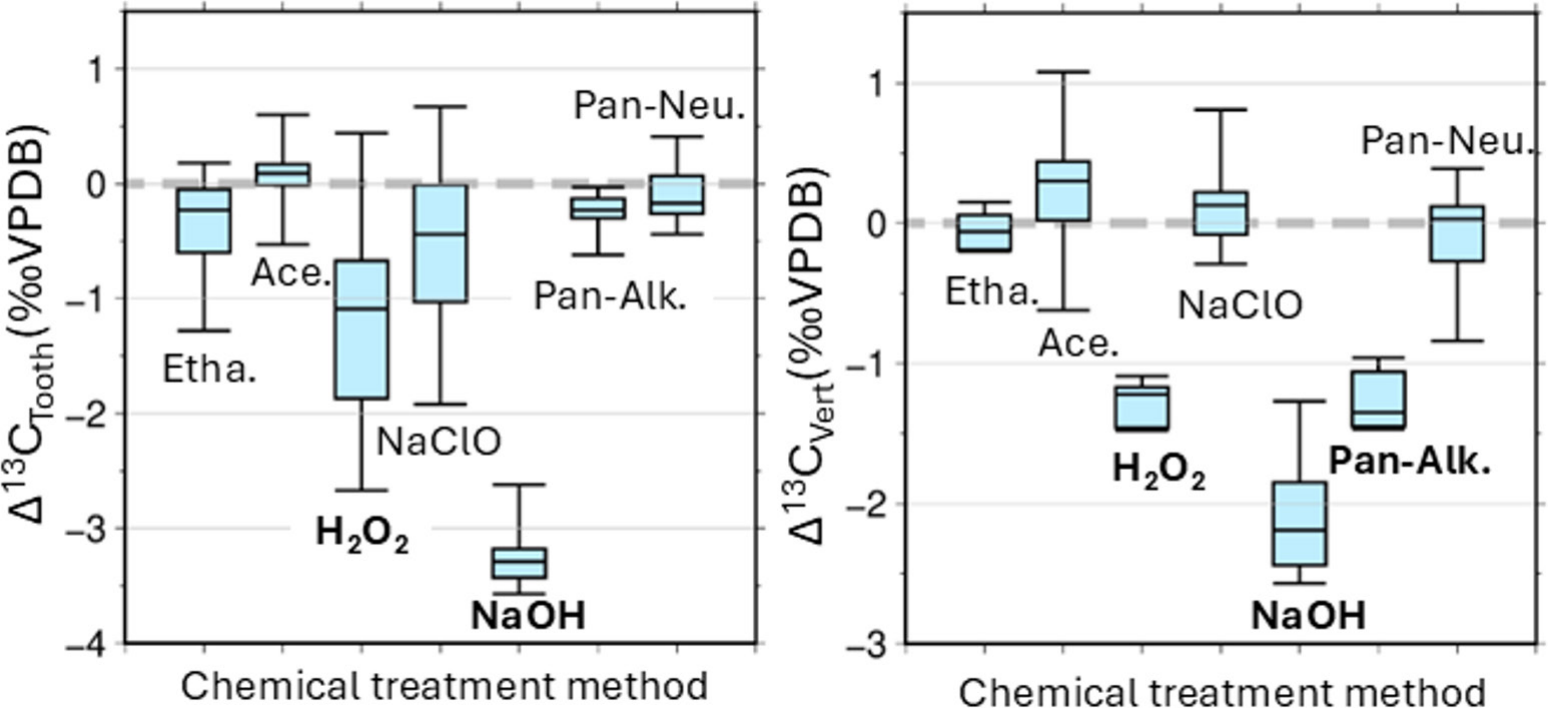
Changes in δ^13^C values caused by chemical treatment. Changes in δ^13^C values of tooth (left graph) and vertebrae (right graph) samples after chemical treatment with each reagent are shown as box plots. The reagent used for each treatment is indicated near the corresponding plot. (Etha: Ethanol, Ace: Acetone, H_2_O_2_: H_2_O_2_ aq., NaClO: NaClO aq., NaOH: NaOH aq., Pan‐Alk.: Alkaline Pancreatin aq., Pan‐Neu.: Neutral Pancreatin aq.) A value of 0 on the vertical axis indicates no change in δ^13^C. Treatments that caused statistically significant decreases exceeding analytical error are shown in bold.

### 3.4 δ^18^O Values

δ^18^O values (‰VPDB) of the tooth samples were not affected by the treatments with organic solvents and acidic solutions but decreased significantly by the treatments with alkaline and pancreatin solutions. The addition of the muscle samples also did not change δ^18^O values significantly (the difference is −0.25‰, *p* < 0.01, *n* = 42, Table S2). The δ^18^O values of carbonate in the samples treated with ethanol or acetone did not decrease significantly from that in samples subjected to physical cleaning, of both teeth and vertebrae (See Figure 4, Figure S8, and Table 1). In contrast, samples treated with NaOH aq. showed a significantly lower δ^18^O compared to physically cleaned samples (Δ^18^O_Tooth_ = −2.32‰ ± 0.42‰, *p* < 0.01, *n* = 20; Δ^18^O_Vert_ = −4.25‰ ± 1.32‰, *p* < 0.001, *n* = 28) (Figure 4). Samples treated with alkaline pancreatin solution exhibited a moderate decrease in δ^18^O relative to physically cleaned samples (Δ^18^O_Tooth_ = −1.11‰ ± 0.49‰, *p* < 0.01, *n* = 20; Δ^18^O_Vert_ = −1.92‰ ± 0.36‰, *p* < 0.001, *n* = 29) (Figure 4). Similarly, samples treated with neutral pancreatin solution also exhibited a moderate decrease in δ^18^O relative to physically cleaned samples (Δ^18^O_Tooth_ = −0.95‰ ± 0.29‰, *p* < 0.01, *n* = 20; Δ^18^O_Vert_ = −0.73‰ ± 0.41‰, *p* < 0.001, *n* = 30) (Figure 4). In samples treated with H_2_O_2_ aq., a significant decrease in δ^18^O was observed in the vertebra (Δ^18^O_Tooth_ = −0.87‰ ± 0.26‰, *p* < 0.001, *n* = 26), whereas a small change was found in the tooth (Δ^18^O_Vert_ = −0.35‰ ± 0.47‰, *p* = 0.013, *n* = 26) (Figure 4). In contrast, in samples treated with NaClO aq., a significant decrease in δ^18^O was observed in the tooth (Δ^18^O_Tooth_ = −3.56‰ ± 1.28‰, *p* < 0.01, *n* = 24), whereas little change was found in the vertebra (Δ^18^O_Vert_ = −0.26‰ ± 1.20‰, *p* = 0.34, *n* = 28) (Figure 4).

**FIGURE 4:**
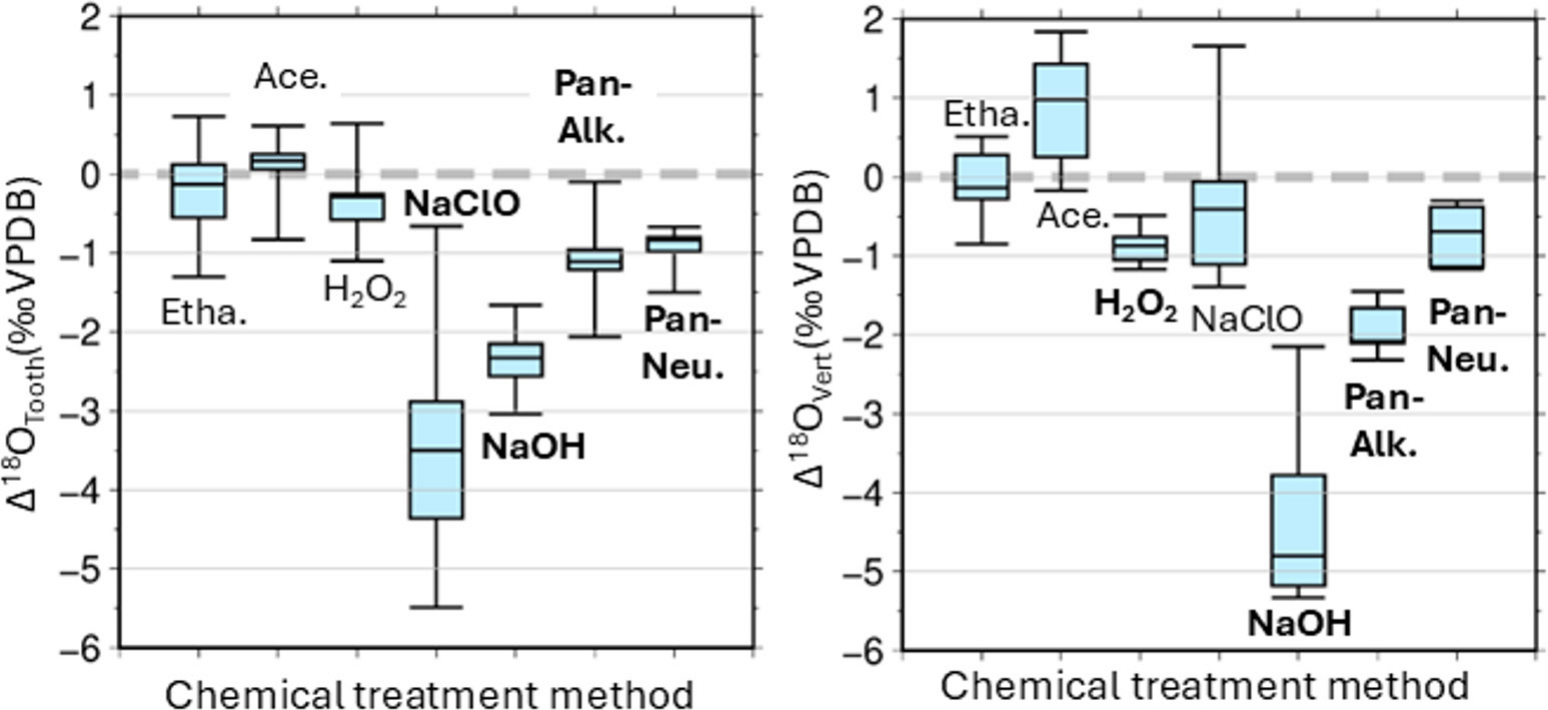
Changes in δ^18^O values caused by chemical treatment. Changes in δ^18^O values of tooth (left graph) and vertebrae (right graph) samples after chemical treatment with each reagent are shown as box plots. The reagent used for each treatment is indicated near the corresponding plot. (Etha.: Ethanol, Ace.: Acetone, H_2_O_2_: H_2_O_2_ aq., NaClO: NaClO aq., NaOH: NaOH aq., Pan‐Alk.: Alkaline Pancreatin aq., Pan‐Neu.: Neutral Pancreatin aq.) A value of 0 on the vertical axis indicates no change in δ^18^O. Treatments that caused statistically significant decreases exceeding analytical error are shown in bold.

### 3.5 XRD Patterns

Figure 5 shows the XRD patterns of the samples subjected to each pretreatment. All tooth samples that underwent the chemical treatments evaluated in this study exhibited XRD patterns similar to those of physically cleaned samples (Figure 5). The 2*θ* angles and relative intensities of the peaks matched those of apatite. Also, the diffraction peaks in all the patterns were broad and largely overlapped. The apatite diffraction peaks obtained in this experiment had low resolution, making it impossible to distinguish between hydroxyapatite and fluorapatite. For the isolated peak around 2*θ* = 26°, no difference in peak width (FWHM) due to pretreatment was observed (Table S5), and no changes in crystallite size or lattice strain attributable to the pretreatments were indicated.

**FIGURE 5:**
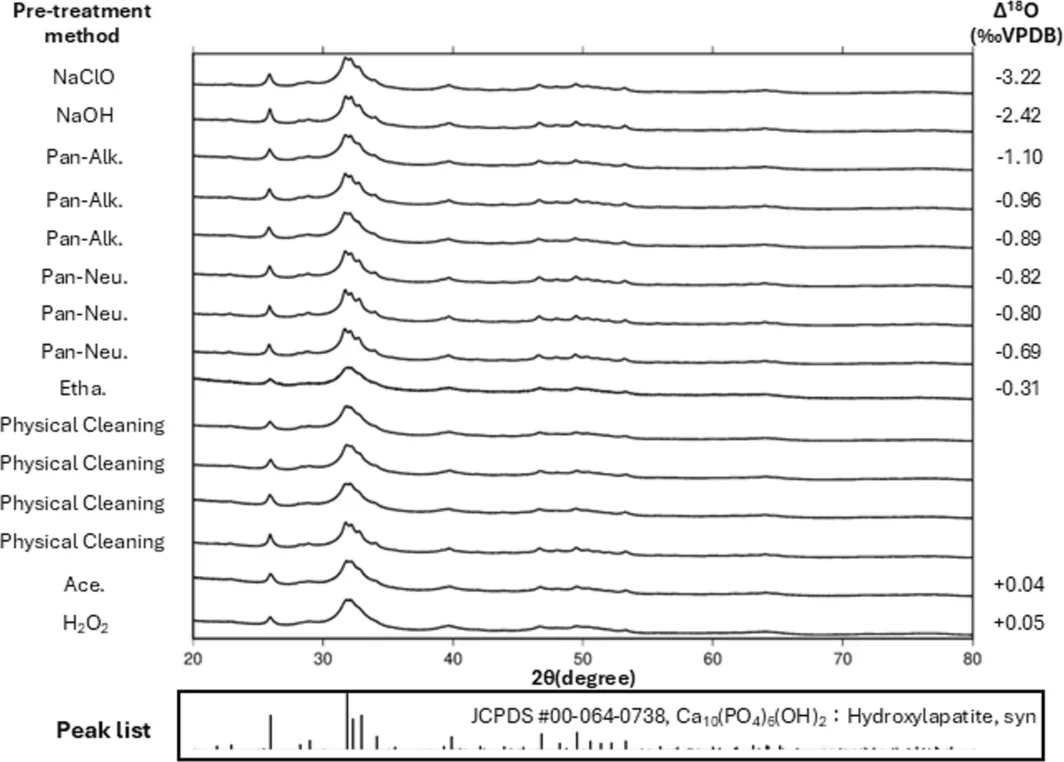
XRD patterns. To the left of each pattern, the reagents used for chemical treatment are shown (Etha.: Ethanol, Ace.: Acetone, NaClO: NaClO aq., NaOH: NaOH aq., Pan‐Alk.: Alkaline pancreatin aq., Pan‐Neu.: Neutral pancreatin aq.). On the right side, the values of δ^18^O change (Δ^18^O, ‰ VPDB) confirmed in this study are also indicated. The lower part of Figure 5 shows the positions and relative intensities of the peaks calculated for hydroxyapatite (JCPDS #00‐064‐0738).

## 4 Discussion

### 4.1 Evaluation of the Isotopic Composition of Samples With Physical Cleaning

The samples subjected to physical cleaning are expected to retain the original isotopic composition of structural carbonate or at least exhibit minimal alteration. No CO_2_ peak was detected from the muscle powder, indicating that organic matter that adheres to the teeth, mainly muscle tissue, produces negligible amounts of CO_2_ as a contaminant gas during the analytical procedure. Also, the isotope analysis showed that no or small differences were detected between the tooth samples and the “tooth + muscle” samples (The difference of δ^13^C: −0.04, *p* = 0.17; the difference of δ^18^O: −0.25, *p* < 0.01; see Table S2). The addition of the muscle samples did not affect the isotopic analysis. Based on these results, we concluded that the isotopic compositions of the mechanically treated samples, despite the greater amount of adhering organic matter (Figure 1 and Figure S4), most likely represent the primary isotopic signatures.

The carbonate content of these samples was 2.60 ± 0.59 wt.% (Figure S6a), lower than the ~3.4 wt.% reported by Elliot et al. [49]. This suggests that the CO_2_ gas derived from residual organic matter after physical cleaning was sufficiently low and that the CO_2_ gas analyzed was predominantly derived from structural carbonate. Therefore, we regarded the carbonate content (wt.%) and isotopic composition of the samples subjected to physical cleaning as representing primary values, making them a suitable reference for assessing isotopic changes induced by subsequent treatments.

### 4.2 Appropriate Pretreatments for Isotope Analysis of Structural Carbonate in Dentin

Treatments with neutral pancreatin solutions can be recommended as appropriate organic matter removal methods for analyzing the δ^13^C of structural carbonate in dentin. In this study, this treatment effectively removed all adhesive muscles (Figure 1 and Figure S4) while preserving the primary δ^13^C values (Figure 3). The crystallite size or lattice strain of the teeth did not change significantly in the XRD patterns (Figure 5), and the amount of carbonate (wt.%) changed little with this treatment (ΔCO_3Tooth_ = 0.40 ± 0.24 wt.%, *p* = 0.002, Figure 2), indicating that the original isotope compositions were likely preserved. Pancreatin contains a variety of enzymes, and its hydrolytic action on organic matter resembles that of the early stages of the natural decomposition process [19]. Wang and Cerling [25] indicated that the δ^13^C of structural carbonate is resistant to alteration during fossilization (diagenesis), including the decay process of organic matter. The minimal effect of organic matter hydrolysis on δ^13^C observed here, consistent with those findings, supports the conclusion that pancreatin treatment is suitable for removing organic matter from dentin prior to δ^13^C analysis of structural carbonate, thereby eliminating potential analytical inference.

Treatment with alkaline pancreatin solution may alter the δ^13^C of structural carbonate owing to differences in the organic matter arrangement within phosphate tissues. In this study, the δ^13^C of dentin‐based tooth samples showed a small change (Δ^13^C_Tooth_ = −0.26‰ ± 0.19‰, *p* = 0.002), whereas the δ^13^C of vertebral samples composed of bone decreased significantly by more than 1‰ (Table S3). This difference may be attributable to the distinct cell distributions in the dentin and bone. Although both are composed of hydroxyapatite, bone contains numerous cells distributed from the center to the periphery [50], whereas dentin contains cells primarily in the vascular region near the tooth center [51]. Treatment with alkaline pancreatin solution may have a smaller effect on the δ^13^C owing to little organic matter in the outer part of the dentin, resulting in smaller changes in the δ^13^C of tooth samples; thus neutral condition is recommended for use.

We recommend the use of a neutral pancreatin solution for the removal of organic matter, as it is the most effective method for δ^18^O analysis of the structural carbonates evaluated in this study. However, because this treatment may cause slight δ^18^O shifts (Δ^18^O), appropriate correction should be applied to obtain more accurate values. In this study, treatment with a neutral pancreatin solution removed all adhesive muscles (Figure 1 and Figure S4) and caused only a minor constant change in δ^18^O (Δ^18^O_Tooth_ = −0.95‰ ± 0.29‰, *p* = 0.0020, Figure 4). The constant Δ^18^O indicates that such changes can be corrected, for example, by using samples subjected to physical cleaning as references and by continually monitoring the Δ^18^O caused by the treatment. In the case of half‐smooth golden pufferfish, the specimen of this study, and where the mean Δ^18^O is approximately −0.95‰, the original δ^18^O value can be reconstructed by adding 0.95‰ to the measured δ^18^O after this treatment. In contrast, Pellegrini and Snoeck [52] recommended treatment with hydrazine; however, the Δ^18^O values for dentin samples were highly variable (−6‰ to 0‰) [52], making correction difficult. In comparison, our treatment with neutral pancreatin solution showed much lower variation (−1.5‰ to 0.5‰), allowing accurate correction. Also, although physical cleaning is likely to keep the isotopic composition of structural carbonate, it cannot be recommended as an ideal treatment when considering sample degradation caused by organic matter and the negative effects on the high‐vacuum and high‐voltage components of the isotope‐ratio mass spectrometer. Thus, the combination of neutral pancreatin pretreatment and systematic Δ^18^O correction provides a reliable approach for obtaining the primary δ^18^O values of structural carbonate, as it also minimizes the negative effects of organic matter on the analysis. When both δ^13^C and δ^18^O of structural carbonate are measured, the original isotopic composition can likely be reconstructed by leaving the δ^13^C values uncorrected and applying corrections only to the δ^18^O values.

Treatment with H_2_O_2_ aq. effectively removed organic matter (Figure 1 and Figure S4) and did not change δ^18^O largely (Δ^18^O_Tooth_ = −0.53‰ ± 0.75‰, *p* = 0.034, Figure 4) but significantly decreased δ^13^C by more than 1‰ (Figure 3). Because the carbon in structural carbonate is bound to hydroxyapatite [11], the substitution of carbon is likely accompanied by the substitution of oxygen. Therefore, a change in δ^13^C suggests that the measured δ^18^O was not primary. Based on this reasoning, we conclude that the treatment with H_2_O_2_ aq. did not preserve the original δ^18^O values. Because the pH of the H_2_O_2_ aq. used in this study was 3, we considered the possibility that acidic conditions partially dissolved the structural carbonate, thereby affecting its isotopic composition. Pellegrini and Snoeck reported partial dissolution of the surface of carbonate biominerals and the changes in both δ^13^C and δ^18^O [52]. However, in our study, no change in carbonate content was observed in the samples treated with H_2_O_2_ aq. One possible explanation is that oxidation by H_2_O_2_ consumed hydrogen ions, increasing the pH and partial reprecipitation of the dissolved carbonate. If so, the carbonate content may not have been changed whereas the original isotopic composition was not fully recovered.

### 4.3 Inappropriate Pretreatments for Isotope Analysis of Structural Carbonate in Dentin

Conventional pretreatment methods developed for isotope analysis of phosphate and collagen in teeth typically employ chemical reagents, such as H_2_O_2_ aq., NaClO aq., and NaOH aq. Although these methods are widely used without concern in studies reconstructing salinity and temperature from phosphate δ^18^O [9, 53], or inferring trophic level and metabolism from collagen δ^13^C [16, 54], they can alter the isotopic composition of structural carbonate [26, 51]. This point is critical because integrating δ^18^O of phosphate and δ^13^C of collagen with the isotopic composition of structural carbonate offers strong potential for distinguishing diagenetic alterations from ecological and physiological signals [55, 56, 57]. Our results demonstrate that these commonly used pretreatment reagents can modify the isotopic compositions of structural carbonates. Therefore, isotopic analysis of structural carbonate should be performed prior to any chemical pretreatment with these reagents, as shown in (1) and (2).

1. H_2_O_2_ aq. and NaClO aq. are commonly used for organic matter removal in the isotopic analysis of phosphate in teeth [58, 59], but they are inappropriate for the isotopic analysis of structural carbonates in dentin. In this study, δ^13^C was significantly lowered by treatment with H_2_O_2_ aq. (Figure 3). Although the accompanying change in δ^18^O was small (Figure 4), oxygen in structural carbonates was likely substituted during the treatment [11], suggesting that the δ^18^O values do not reflect their original state. The samples subjected to the treatment with NaClO aq. increased the variance of δ^13^C (Figure 3), significantly decreased δ^18^O (Figure 4), and dramatically increased carbonate content (wt.%; Figure 2), indicating the formation of secondary carbonates. Similar alterations to carbonate isotopic composition from these treatments have been reported previously [16, 26, 52]. These results indicate that H_2_O_2_ and NaClO treatments can alter δ^13^C and δ^18^O of tooth carbonate, rendering the samples unsuitable for isotope analysis of structural carbonates.
2. NaOH is commonly used for collagen extraction from bioapatite tissue [39, 60] but is inappropriate for isotopic analysis of structural carbonate in dentin. In this study, NaOH treatment substantially decreased both δ^13^C and δ^18^O of structural carbonate (Figures 3 and 4) and markedly increased carbonate content (wt.%; Figure 2), indicating the incorporation of secondary carbonate. Similar effects have been reported for vertebral carbonates of ray‐finned fish [16], consistent with our results. Therefore, NaOH treatment should be avoided for isotope analysis of structural carbonate.

### 4.4 Implication for Early Diagenesis

Treatment with a neutral pancreatin solution slightly altered the δ^18^O of structural carbonate, suggesting that the organic decay process could disturb its original isotopic composition. In our study, δ^18^O decreased by approximately 1‰ following pancreatin treatment (Figure 4). Pancreatin contains digestive enzymes such as amylases, proteases, and lipases, which hydrolyze organic matter in a reaction similar to the organic decomposition process during fossilization [19, 61]. In the early stage of diagenesis, the initial step in organic matter decomposition is enzymatic hydrolysis [19]. At this step, anaerobic bacteria secrete various enzymes to hydrolyze organic matter into smaller units [19]. Given this similarity, it is plausible that the δ^18^O of structural carbonate in fossil dentin does not preserve a primary biogenic signal, such as temperature and salinity. This hypothesis is consistent with the report that the isotope composition of structural carbonate was altered even in relatively new Holocene fossil dentin [26]. Although several previous studies have analyzed the δ^18^O of structural carbonate and clumped isotope composition in the fossil dentin [62, 63], the δ^18^O of structural carbonate in fossil dentin may be diagenetically altered during the early organic matter decomposition process. Therefore, δ^18^O of structural carbonate in fossil dentin should be carefully treated as a proxy for environmental and ecological reconstructions.

## 5 Conclusion

We developed a method for removing organic matter from dentin in extant ray‐finned fish teeth for isotopic analysis of structural carbonate. The treatments with neutral or alkaline pancreatin solution are recommended as effective methods for removing organic matter while preserving the original δ^13^C value of structural carbonate. However, no method has been identified that removes organic matter without changing the δ^18^O of structural carbonate. The treatment with neutral pancreatin solution is the preferred alternative because the change in δ^18^O was relatively small and consistent. Given the reproducibility of this shift, the δ^18^O value obtained after the treatment can be corrected to approximate the primary δ^18^O.

## Author Contributions

**Toshikazu Furuya:** conceptualization, methodology, data curation, investigation, validation, formal analysis, visualization, writing – original draft, writing – review and editing. **Kazuki Yokouchi:** data curation, investigation, funding acquisition, resources, writing – review and editing. **Taiga Okumura:** methodology, resources, writing – review and editing. **Kotaro Shirai:** conceptualization, methodology, supervision, funding acquisition, project administration, resources, writing – review and editing.

## Supporting information


**Figure S1:** rcm70200‐sup‐0001‐Figure_S1.TIF. *Lagocephalus spadiceus* (Richardson, 1845) The specimens of *L. spadiceus* were immediately frozen at −25°C after capture. To extract teeth and vertebrae, they were thawed, and the photograph shown in Figure S1 was taken.

**Figure S2:** Optical microscope image of a cross section of the tooth. The tips of the teeth were observed under transmitted light. The red arrows indicate the enameloid layers.

**Figure S3:** Sampling area. The area enclosed by the red line indicates the stations where specimens were captured. The gray area represents land. The blue area on the map represents the sea, with depth contours drawn at 10‐m intervals and shading indicating water depth. The bathymetric data were obtained from NOAA's ETOPO1. The latitude and longitude of each sampling station were referenced from Sone et al. (2022), Figure 2.

**Figure S4A:** “Before” and “After” treatment photos. The teeth shown here have undergone each pretreatment. From left to right, the photographs show the samples before treatment, after treatment, and after drying. The scale bar represents 1 cm. All the samples were photographed from the labial side. Both left and right, upper and lower jaw teeth are included. The photograph of the upper jaw includes the maxilla bones.

**Figure S4B:** “Before” and “After” treatment photos.

**Figure S5:** Appearance of vertebrae samples after pretreatment. The vertebrae shown here have undergone each pretreatment. All the samples were photographed from the left side. Vertebrae from various positions, ranging from cervical to caudal, are included.

**Figure S6:** Changes in carbonate content caused by chemical treatments. In graphs (a), (b), (c), and (d), the horizontal axis shows the specimen number or sample‐group number. Above each graph is the reagent used for the chemical treatment (Etha.: ethanol, Ace.: acetone, H_2_O_2_: hydrogen peroxide, NaOH: sodium hydroxide solution, Pan‐Alk.: alkaline pancreatin solution, Pan‐Neu.: neutral pancreatin solution). In (a) and (c), white symbols indicate samples that underwent physical removal only, whereas black symbols indicate samples that underwent chemical treatment. (a) Carbonate content (wt.%) of each tooth sample. Because we set paired treated and untreated samples for both upper and lower teeth for each individual, there are four data points per specimen. (b) Change in carbonate content caused by each chemical treatment applied to the tooth samples. (c) Carbonate content (wt.%) of vertebral samples. For each individual, all vertebrae subjected to the same chemical treatment were grouped as one sample group and compared to a group of vertebrae from the same individual that experienced only physical removal. (d) Change in carbonate content for each chemically treated vertebra group, plotted as the difference between the mean carbonate content of the chemically treated group and that of the physically cleaned group from the same individual.

**Figure S7:** Changes in carbonate δ^13^C induced by chemical treatments. In graphs (a), (b), (c), and (d), the horizontal axis shows the specimen number or sample‐group number. Above each graph is the reagent used for the chemical treatment (Etha.: ethanol, Ace.: acetone, H_2_O_2_: hydrogen peroxide, NaOH: sodium hydroxide solution, Pan‐Alk.: alkaline pancreatin solution; Pan‐Neu.: neutral pancreatin solution). In (a) and (c), white symbols indicate samples that underwent physical removal only, whereas black symbols indicate samples that underwent chemical treatment. (a) δ^13^C (‰VPDB) of structural carbonate of each tooth sample. Because we set paired treated and untreated samples for both upper and lower teeth for each individual, there are four data points per specimen. (b) Change in δ^13^C (‰VPDB) caused by each chemical treatment applied to the tooth samples. (c) δ^13^C (‰VPDB) of vertebral samples. For each individual, all vertebrae subjected to the same chemical treatment were grouped as one sample group and compared to a group of vertebrae from the same individual that experienced only physical removal. (d) Change in δ^13^C (‰VPDB) for each chemically treated vertebra group, plotted as the difference between the mean δ^13^C (‰VPDB) of the chemically treated group and that of the physically cleaned group from the same individual.

**Figure S8:** Changes in carbonate δ^18^O induced by chemical treatments. In graphs (a), (b), (c), and (d), the horizontal axis shows the specimen number or sample‐group number. Above each graph is the reagent used for the chemical treatment (Etha.: ethanol, Ace.: acetone, H_2_O_2_: hydrogen peroxide, NaOH: sodium hydroxide solution, Pan‐Alk.: alkaline pancreatin solution, Pan‐Neu.: neutral pancreatin solution). In (a) and (c), white symbols indicate samples that underwent physical removal only, whereas black symbols indicate samples that underwent chemical treatment. (a) δ^18^O (‰VPDB) of structural carbonate of each tooth sample. Because we set paired treated and untreated samples for both upper and lower teeth for each individual, there are four data points per specimen. (b) Change in δ^18^O (‰VPDB) caused by each chemical treatment applied to the tooth samples. (c) δ^18^O (‰VPDB) of vertebral samples. For each individual, all vertebrae subjected to the same chemical treatment were grouped as one sample group and compared to a group of vertebrae from the same individual that experienced only physical removal. (d) Change in δ^18^O (‰VPDB) for each chemically treated vertebra group, plotted as the difference between the mean δ^18^O (‰VPDB) of the chemically treated group and that of the physically cleaned group from the same individual.

**Table S1:** Tooth sample analysis results.
**TableS2:** Tooth and muscle sample analysis results.
**Table S3:** Supporting information.
**Table S4:** Analysis error.
**Table S5:** XRD diffraction pattern.

## Data Availability

The data that supports the findings of this study are available in the supplementary material of this article.
